# Supplementary material for: Amphiphilic Nucleobase-Containing Polypeptide Copolymers—Synthesis and Self-Assembly
Source: Polymers (Basel). 2020 Jun 16;12(6):1357. doi: 10.3390/polym12061357 (PMC7362222; doi:10.3390/polym12061357)
Supplement: Supplementary file 1 [file polymers-12-01357-s001.pdf]

Amphiphilic nucleobase-containing polypeptide copolymers: synthesis and self-assembly

Michel NGuyen[1], Khalid Ferji,[2] Sébastien Lecommandoux,[3] Colin Bonduelle[1,3]*

Dr. M. NGuyen; CNRS, LCC (Laboratoire de Chimie de Coordination (UPR8241)
205 route de Narbonne, F-31077 Toulouse, France.
E-mail: michel.nguyen@lcc-toulouse.fr.
Dr. K. Ferji; Univ. Lorraine, CNRS, LCPM, F-54000 Nancy, France.
E-mail: khalid.ferji@univ-lorraine.fr
Pr. S. Lecommandoux, Dr. C. Bonduelle; Univ. Bordeaux, CNRS, Bordeaux INP, LCPO, UMR 5629, F-33600, Pessac, France.
E-mail: lecommandoux@enscbp.fr, colin.bonduelle@enscbp.fr.

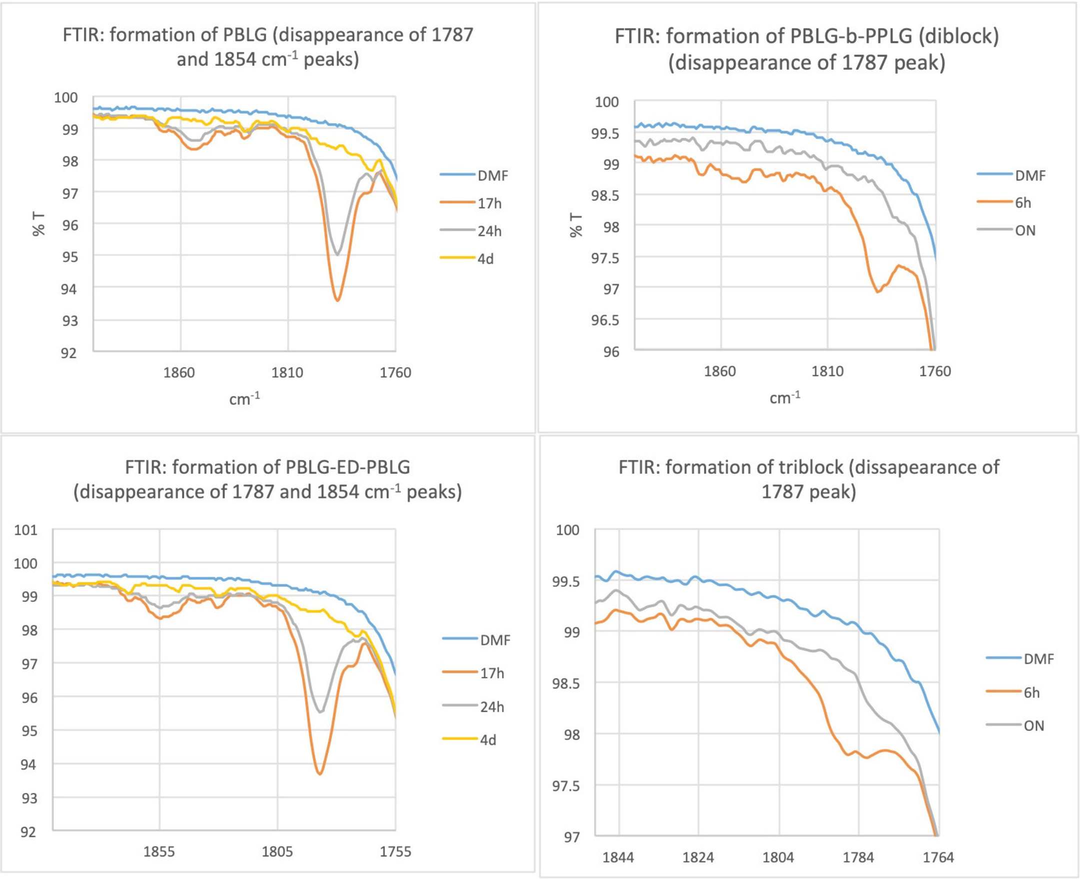

Figure S1. The ring-opening polymerizations were monitored by FTIR following the disappearance of the NCA peaks in DMF (ON: overnight)

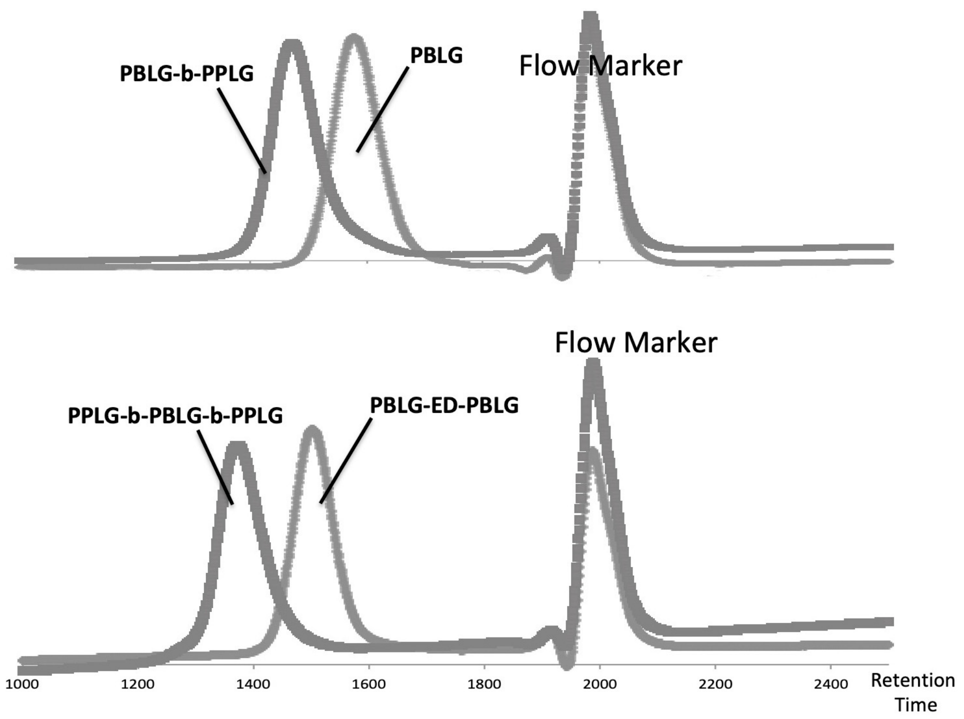

Figure S2. Characterizations of the polypeptides backbones before click by SEC analysis in DMF (1% LiBr). RI detection (flow marker: toluene).

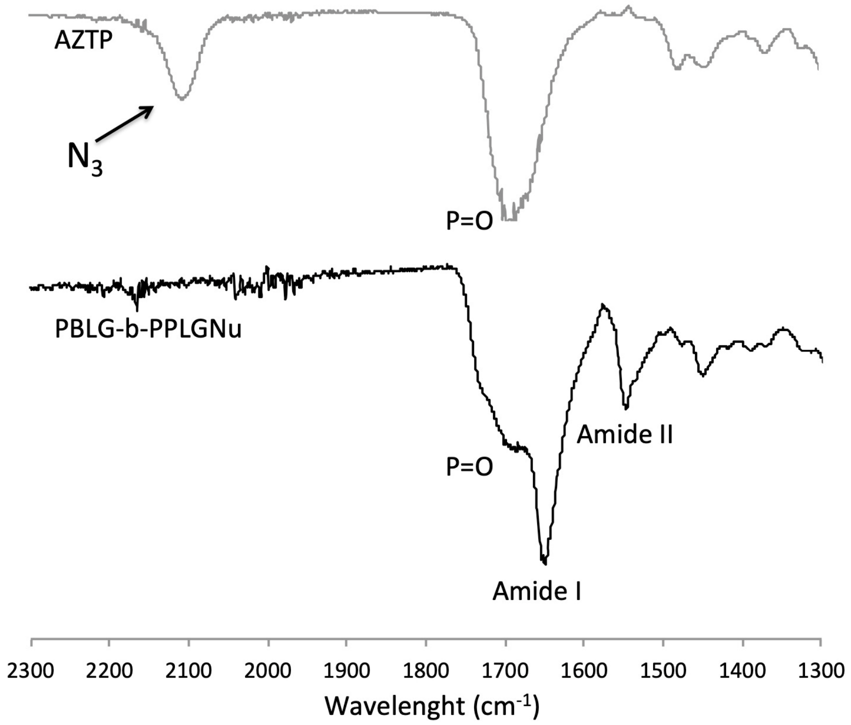

Figure S3. FTIR spectra of PBLG-b-PPLGNu (powder). Comparing this spectrum to FTIR spectroscopy analysis of AZTP clearly indicates that azide peak disappeared.

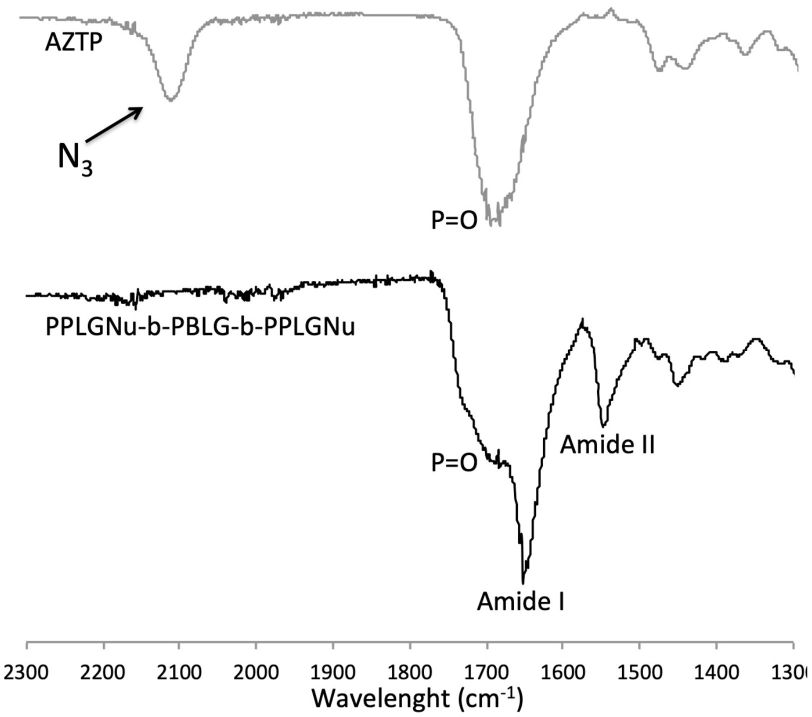

Figure S4. FTIR spectra of PPLGNu-b-PBLG-b-PPLGNu (powder). Comparing this spectrum to FTIR spectroscopy analysis of AZTP clearly indicates that azide peak disappeared.

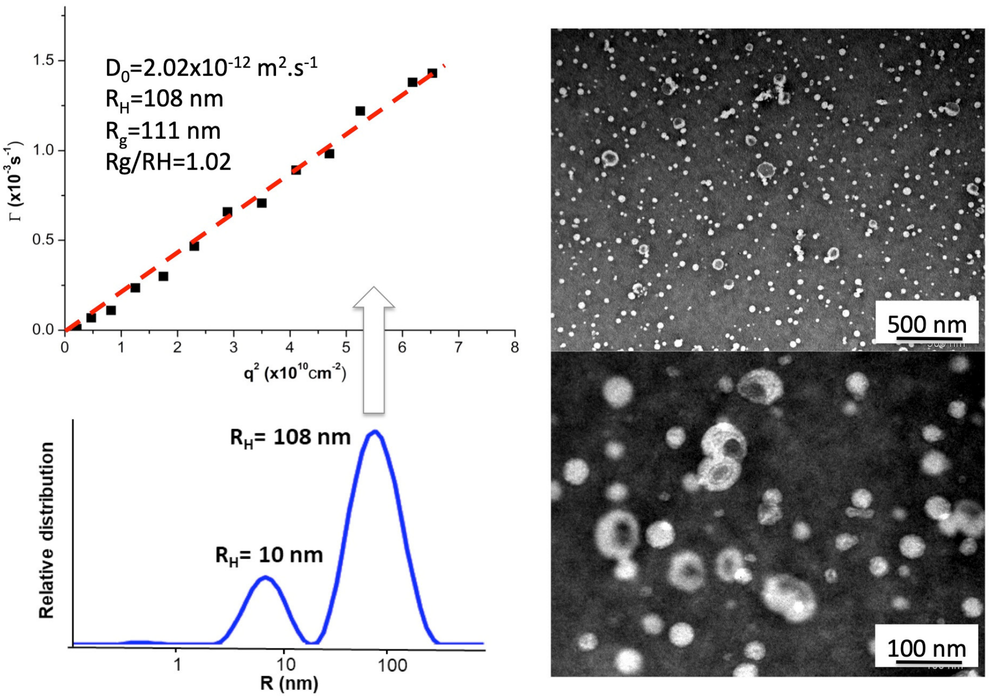

Figure S5. Vesicles based on PPLGNu-b-PBLG-b-PPLGNu triblock copolypeptide: left) Multi-angle light scattering experiments performed to calculate the ***ρ***-ratio. At high angles, another population of small spherical objects are observed; right) Different TEM imaging evidencing the hollow structure of the nanoobjects.
